# Podokinetic After-Rotation Is Transiently Enhanced or Reversed by Unilateral Axial Muscle Proprioceptive Stimulation

**DOI:** 10.1155/2019/7129279

**Published:** 2019-03-11

**Authors:** Stefania Sozzi, Antonio Nardone, Oscar Crisafulli, Marco Schieppati

**Affiliations:** ^1^Centro Studi Attività Motorie (CSAM), Istituti Clinici Scientifici Maugeri IRCCS, Pavia, Italy; ^2^Dipartimento di Scienze Clinico-Chirurgiche, Diagnostiche e Pediatriche, Pavia, Italy; ^3^Istituti Clinici Scientifici Maugeri IRCCS, Pavia, Italy; ^4^Dipartimento di Neuroscienze (DINOGM), Università di Genova, Italy; ^5^LUNEX International University of Health, Exercise and Sports, Differdange, Luxembourg

## Abstract

Unilateral axial muscle vibration, eliciting a proprioceptive volley, is known to incite steering behavior. Whole-body rotation while stepping in place also occurs as an after-effect of stepping on a circular treadmill (podokinetic after-rotation, PKAR). Here, we tested the hypothesis that PKAR is modulated by axial muscle vibration. If both phenomena operate through a common pathway, enhancement or cancellation of body rotation would occur depending on the stimulated side when vibration is administered concurrently with PKAR. Seventeen subjects participated in the study. In one session, subjects stepped in place eyes open on the center of a platform that rotated counterclockwise 60°/s for 10 min. When the platform stopped, subjects continued stepping in place blindfolded. In other session, a vibratory stimulus (100 Hz, 2 min) was administered to right or left paravertebral muscles at lumbar level at two intervals during the PKAR. We computed angular body velocity and foot step angles from markers fixed to shoulders and feet. During PKAR, all subjects rotated clockwise. Decreased angular velocity was induced by right vibration. Conversely, when vibration was administered to the left, clockwise rotation velocity increased. The combined effect on body rotation depended on the time at which vibration was administered during PKAR. Under all conditions, foot step angle was coherent with shoulder angular velocity. PKAR results from continuous asymmetric input from the muscles producing leg rotation, while axial muscle vibration elicits a proprioceptive asymmetric input. Both conditioning procedures appear to produce their effects through a common mechanism. We suggest that both stimulations would affect our straight ahead by combining their effects in an algebraic mode.

## 1. Introduction

When normal subjects step in place eyes closed, they rarely maintain their initial straight-ahead orientation. Most subjects slowly deviate or translate from the initial stepping spot without being aware of it. The rotation velocity is normally modest, so that little more than a few dozen degrees are to be expected within one minute time [1]. Several researchers have tried to identify the factors responsible for the changes in body orientation occurring while stepping on the spot. However, poor correlations exist between leg length difference, handedness, or lateral preference, while head posture increases the error [2–4]. This suggests a role for the neck proprioceptive or vestibular input in stabilizing the stepping orientation [5, 6].

Rotation can be definitely larger in patients with vestibular lesions. The stepping in place task had been introduced many decades ago as a clinical test [7], and it is still used nowadays, because it can add to the overall clinical picture and may be suggestive of a labyrinthine lesion. This test alone is clearly insufficient to establish a diagnosis though, because of its ample variability within and across subjects and patients [8]. However, it can indicate orientation biases in patients with neck dystonia [9, 10]. This is in keeping with the evidence that neck muscle activity interferes with the control of body orientation during stepping in place [11–13].

Body rotation while stepping in place can be experimentally elicited, as for instance by gaze redirection [14]. Proprioception can also powerfully stimulate steering behavior while stepping in place or walking. Vibration of the lateral neck muscles definitely produces a rotation toward the opposite side (vibration of the right sternocleidomastoid makes the body rotate to the left or counterclockwise) [1, 9]. A similar effect is induced by vibration of other axial muscles, like the trunk paraspinal muscles [15], but not of limb muscles [16].

Another elegant way of producing whole-body rotation while stepping (eyes closed) is to preliminarily have subjects stepping eyes open on the center of a motorized circular treadmill for a while maintaining a constant orientation in space and ask them to continue stepping in place eyes closed on the still treadmill. The stepping period on the platform is called podokinetic stimulation (PKS), and the ensuing whole-body rotation, unbeknownst to the stepping subject, is called podokinetic after-rotation (PKAR) [17–20]. Interestingly, the very same outcome (stepping and rotating) can be obtained by having subjects stepping in place and deliberately rotating: when asked to continue stepping eyes closed without rotating, the podokinetic after-effect shows up again [20].

Do all these conditioning procedures produce their effect through a common mechanism? Is there some neural center for yaw orientation in space normally accessed by vestibular or proprioceptive input or optokinetic stimulus [21] and be they elicited by disease, stimulation, or voluntary rotating behavior [22]? Here, we tested the hypothesis that the podokinetic after-effect can be enhanced by asymmetric proprioceptive stimulation in the form of unilateral axial muscle vibration.

## 2. Methods

### 2.1. Subjects and Tasks

Seventeen young healthy subjects (6 males and 11 females) participated in this study. Their mean ± SD age, height, and weight were 28.2 ± 7.2 yrs, 173.2 ± 10.8 cm, and 63.9 ± 13.2 kg, respectively. Experiments were performed after the adequate understanding and written informed consent of each subject. The ethics committee of the Istituti Clinici Scientifici Maugeri had approved the experiments (approval number 806 CEC).

Subjects took part in three different experimental sessions in three different days, at least one week apart. Before the sessions, subjects familiarized with the task, by stepping in place eyes closed for one minute. Within this period, changes in heading direction never exceeded ±90 deg of body yaw rotation (mean body rotation: 18.8 ± 53.4 deg), which was the upper limit of spontaneous rotation for including subjects in the study.

**Figure 1:**
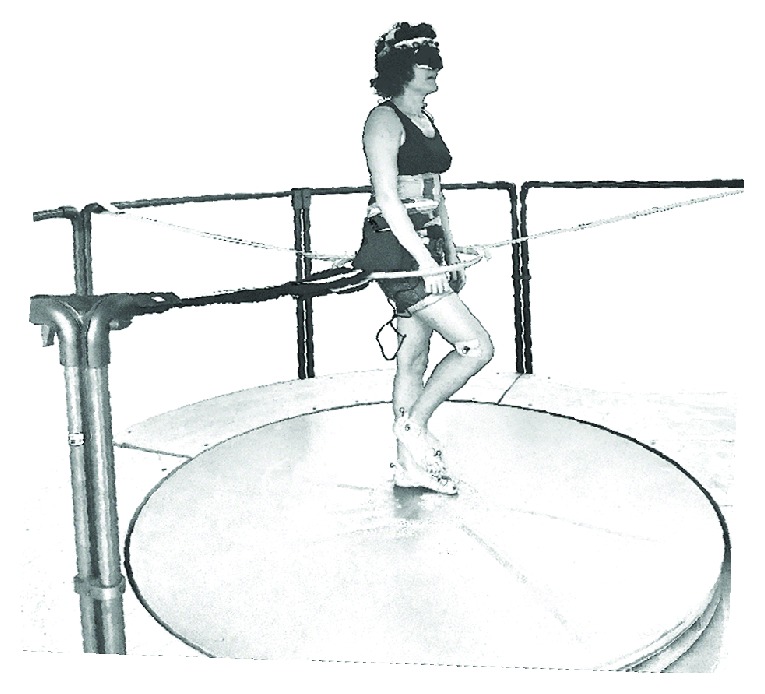
Rotating platform. Subjects stepped in place at the center of a platform that rotated counterclockwise at a velocity of 60 deg/s. When the platform stopped, they continued stepping in place blindfolded. In order to prevent subject's translation from the center of the platform, a lightweight hula hoop was fixed to the platform outer railing by elastic straps at pelvic height.

During the first session, subjects had to step in place eyes open at their own cadence with bare feet at the center of a round platform of 2 m diameter that rotated counterclockwise at an angular velocity of 60°/s for 10 min. This procedure represents the podokinetic stimulation (PKS). A ten-minute duration for the PKS has been shown to be sufficient [20, 23] for inducing a clear-cut podokinetic after-effect (PKAR), consisting in involuntary whole-body rotation while stepping in place on a firm support base (i.e., the same platform, motionless) without vision. This rotation occurs in a direction opposite to the direction of platform rotation (in this case, subjects rotated clockwise, i.e., in the direction of the rotatory effort exerted in order to counteract the platform rotation) [17, 20, 24]. During the PKS period, subject maintained a constant position of the body in space. Their eyes were open, and they were free to look at the laboratory space (they fixed the gaze in front of them to a chosen landmark at about a two-meter distance). After this period, the platform was stopped. Subjects wore an eye mask at the forehead level during the PKS, and when the platform halted, they simply lowered it at the eye level to block vision while they continued stepping in place. In the after-period, the PKAR normally starts and reaches a peak within few seconds [20, 25, 26] and so did in our subjects. The PKAR normally lasts several minutes. In the present study, the recording lasted for 11 min. During both the PKS and PKAR periods, subjects stepped inside a plastic hula hoop of 50 cm of diameter, loosely fixed at pelvic height by elastic straps secured to the platform outer railing (see Figure 1). This was done in order to prevent subjects' translation from the center of rotation of the platform while stepping in place, both during PKS and during the after-period in which subjects were blindfolded. Lightly touching the hula hoop with the pelvis occurred occasionally, but this gave no cue regarding the body rotation sense or the position in space during the PKAR.

In another experimental session, following 10 min of PKS as in the session mentioned above, a vibratory stimulus was administered by means of a muscle vibration device (VB 115, Techno Concept, France) during the after-period of stepping in place onto the still platform (PKAR). This device (a cylinder of 10 cm length and 3 cm diameter) was placed with the long axis horizontal over the belly of the right paravertebral muscles at lumbar level, about 3 cm lateral to 3rd lumbar vertebra [16] and fixed by a large elastic belt. A continuous vibratory stimulation (100 Hz frequency) was administered after the first minute from the instant of platform stop, for a period of 2 minutes. The strength of the stimulation was set to the maximum amplitude of the device (delivering a transversal displacement of 0.85 mm) [27], but the effective strength of the vibratory stimulus was likely affected by differences in the tissue stiffness and lumbar lordosis of the participants. The same 2 min vibratory stimulus was repeated after 2 min from the end of the former so as to yield alternate periods of stepping in place without and with muscle vibration (this condition is referred to as PKAR+right vibration). The signal from a wireless EMG probe (BTS Bioengineering, Milan, Italy) was fixed to the vibration device and its output synchronized with the acquisition of the kinematic data in order to identify the vibratory stimulation periods. Before the PKS, subjects performed a “control” trial in order to assess and measure the effect of right lumbar vibratory stimulation during simple stepping in place blindfolded on the still platform: after a minute of stepping, the vibratory stimulation was triggered and kept for 1 minute.

A further session mirrored the above procedure, except for the position of the vibration device, which was now placed on the left side of the paravertebral muscles at lumbar level (PKAR+left vibration). Also in this case, the PKS was preceded by a “control” trial: after a minute of stepping in place blindfolded on the still platform, the vibratory stimulation was administered to the lumbar muscle of the left side.

The sessions with vibration (left or right) were randomized across subjects. An interval of at least one week elapsed between sessions.

### 2.2. Data Acquisition and Analysis

Reflective markers were placed bilaterally on the following body parts in order to estimate whole-body rotation and feet movement: vertex and lateral head, acromion, lateral malleolus, heel, and forefoot (dorsally, at 1st metatarsal-phalangeal joint). The position in space of these markers was recorded by means of a 12-camera optoelectronic system (Smart D, BTS, Italy) at a sampling frequency of 140 Hz. Offline analysis was performed on the data acquired in a time window that started 2 min before the platform stopped, therefore including the last part of PKS until the end of the PKAR period (9 min from the end of the PKS). The marker traces were filtered with a low-pass filter with a cut-off frequency of 1.5 Hz [20] with a software developed in MATLAB (MathWorks Inc., USA). This cut-off frequency was chosen based on the frequency spectrum of the shoulder traces during whole-body yaw rotation, which showed no content higher than 1.1 Hz frequency.

In order to evaluate body rotation, the angle described in the horizontal plane by the segment defined by the markers placed on the shoulders was calculated by means of a MATLAB software for each trial of each subject. The cumulative angle described by the body during the PKAR (with and without vibration) was calculated as the sum of the successive angles described by the shoulders during the entire acquired epochs. The instantaneous velocity of body rotation was calculated as the derivative of the trace of the cumulative angle. The mean value of shoulder rotation velocity of all the subjects was fitted with the exponential function *y* = *A* + *B*∗*e*^−^^*t*/*τ*1^ + *C*∗*e*^−^^*t*/*τ*2^ [19, 20, 25, 26, 28] using the iterative gradient method of the Excel® Solver Utility. *τ*_1_ and *τ*_2_ were the time constants, *A* was the asymptotic value of the function, and *A* + *B* + *C* was the intercept with the ordinate. The values of these parameters were computed by using the minimum sum squared algorithm. The maximum value of the double exponential function was the peak of rotation velocity during PKAR and was used to estimate the time at which the maximum rotation velocity was reached. The time constant of the decay of the posteffect disappearance was estimated by *τ*_2_.

For each condition and for each subject, the latency of the onset of the vibration effect during the “control” stepping task was defined by the time instant at which the rotation velocity of the shoulders exceeded the mean value ± 1 SD of velocity of the previous period without vibration. In order to estimate the time course of the effects induced by vibration during stepping in place, the trace of shoulder angular velocity was fitted with the exponential function *y* = *A* + *B*∗*e*^−^^*t*/*τ*^ using the iterative gradient method of the Excel® Solver Utility. The same minimum sum squared algorithm allowed to estimate the time constant (*τ*) with which vibration-induced body rotation reached a plateau.

Cadence, stance phase duration, and step yaw angle of the right foot (the foot corresponding to the direction of CW rotation effort during PKAR) were calculated by a software developed in LabVIEW (National Instruments Corporation, Austin, TX, USA). The stance duration was defined by the time interval during which the trace of the malleolus remained below a threshold defined by the lowermost vertical position reached by the marker placed on the malleolus plus 10% of this value. The step yaw angle was calculated (as for the shoulder angle) as the angle described in the horizontal plane by the segment defined by the markers placed on the heel and forefoot. The cumulative angle described by the foot was the sum of the successive angles described during the entire acquired epochs. The angle described during each step was calculated as the difference between the cumulative angle at the beginning of each step (identified by the time at which the trace of the malleolus exceeded the threshold defined above for the analysis of the stance phase duration) and the end of each step.

### 2.3. Statistical Analysis

A 2-way repeated measures ANOVA with experimental condition (PKAR, PKAR+right vibration, and PKAR+left vibration) and intervals (vibration or no vibration) as factors was used to compare body angular rotation velocity, cadence, and foot angle. A 2-way repeated measures ANOVA with side of vibration (right or left) and conditions (vibration during “control” stepping in place, vibration during PKAR) was used to compare the latency from the onset of vibration and onset of the effect of vibration during stepping in place or PKAR. For all ANOVAs, the post hoc test analyses were made with Fisher's LSD test. The software package used was Statistica (StatSoft, USA).

## 3. Results

### 3.1. Vibration during the “Control” Stepping in Place Task

**Figure 2:**
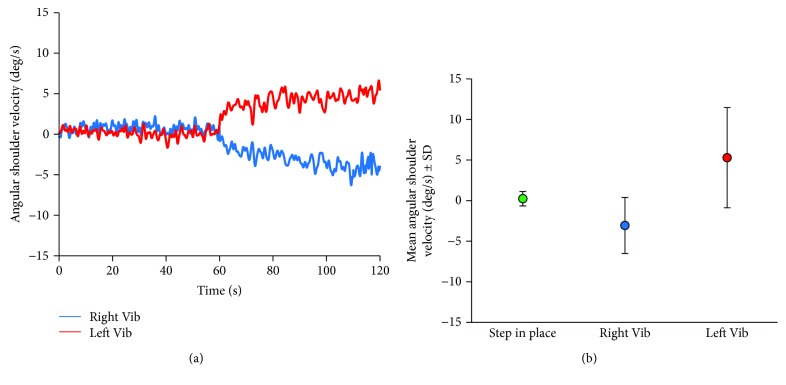
Vibration during stepping in place. (a) Mean body angular velocity. After the onset of the vibratory stimulation (at 60s) of the right lumbar muscle (blue trace), subjects started to rotate counterclockwise (negative values in ordinate). When the vibration was applied to the left side (red trace), subjects rotated clockwise (positive values). (b) Mean angular velocity under stepping in place and right vibration and left vibration conditions. There is no difference in the absolute value of rotation velocity between right and left side vibrations.

Figures 2(a) and 2(b) show the mean rotation angle (average of all subjects' traces) during the simple stepping in place task. Stepping under “control” blindfolded condition, in the absence of vibration, lasted 1 min (from 0 s to 60 s in Figures 2(a)). It was followed by a period of 1 min vibratory stimulation (from 60 s to 120 s). The two traces show the body yaw rotation corresponding to the vibratory stimulation administered to the right (blue trace) or to the left side (red) of the trunk.

During vibration, the angle covered by the 1 min body rotation amounted to 173 deg (right vibration) and to 316 deg (left vibration), corresponding to a mean angular rotation velocity of -3.1 ± 3.4 deg/s and 5.2 ± 6.1 deg/s, respectively (Figure 2(b)). There was ample variability in the rotation across subjects, as shown by the error bars in Figure 2(b). The angular velocity (in absolute value) was not different within subjects during vibration to the left or right side (paired *t*-test, *p* = 0.14).

The onset of body rotation from vibration onset was variable across subjects and ranged between 0.3 s and 20 s. The mean latency of the initial body rotation was 6.0 ± 5.8 s for the right and 5.9 ± 7.9 s for the left vibration stimulation (paired *t*-test *p* = 0.96). Then, rotation continued at a rather constant velocity for the entire period of vibration. All subjects collapsed, a plateau in the exponential trace of the rotation velocity was approached, having a similar time constant (mean of the time constants computed for each subject) for both right (17.8 ± 12.95 s) and left side vibrations (17.6 ± 14.7 s) (paired *t*-test, *p* = 0.97).

### 3.2. Vibration Administered during the Podokinetic After-Rotation

**Figure 3:**
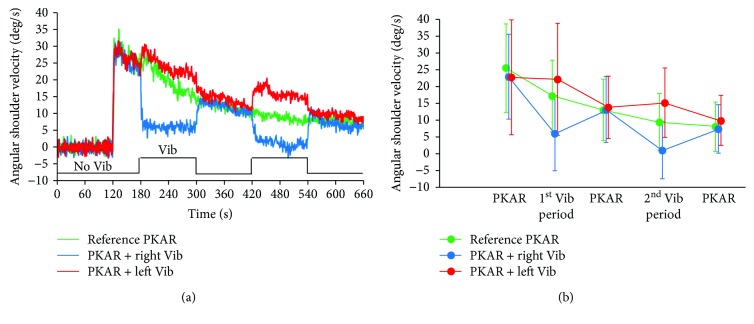
Effects of vibration during PKAR. (a) Mean angular rotation velocity during the PKAR period without vibration (green trace), PKAR with vibration of the lumbar muscle of the right side (blue trace), and PKAR with vibration of lumbar muscle of the left side vibration (red trace). All traces show the last 2 minutes of PKS (0-120 s). During the trials with vibration, the vibration was administered at 180 s and at 420 s and lasted two minutes. (b) Mean angular shoulder velocity averaged during different time intervals: from 150 s to 180 s (PKAR, no vibration), from 220 s to 280 s (1st period of vibration), from 300 s to 360 s (PKAR, no vibration), from 440 s to 500 s (2nd period of vibration), and from 540 s to 600 s (no vibration). During the periods with vibration (Right Vib (blue dots) or Left Vib (red dots)), the mean values of the angular velocity are different than those of the corresponding PKAR (without vibration, green dots) intervals.

The traces of Figure 3(a) show the time course of the PKAR, in the three conditions recorded in the different sessions: PKAR without vibration (reference PKAR, green trace), PKAR with two successive superimposed periods of vibration administered to the right side (blue trace), and PKAR with two vibration periods to the left side (red trace). Each trace is the average of the effects recorded in each subject, separately for the three conditions. All traces start with the two last minutes of PKS (from 0 to 120 s), during which period subjects did not rotate, since they stepped on the rotating platform eyes open and kept their orientation in space. It is also obvious that, on the average, the spontaneous body rotation (PKAR) started very soon after the end of the PKS and reached a maximum value between 7 s and 8 s in all PKAR conditions (reference, 7.46 s; left vibration, 7.07 s; and right vibration, 7.93 s). From this moment, the angular body rotation began to decrease with a time constant of about 3 min (reference PKAR, 176 s; PKAR with right vibration, 178 s; and PKAR with left vibration, 200 s).

The mean latency from vibration onset to initial change in body rotation velocity during PKAR was 12.06 ± 12 s for the first period of right vibration and 9.13 ± 10.1 s for the second period of right vibration. For left-sided vibration, the mean latency to initial change in body rotation velocity was 5.98 ± 6.3 s for the first period of vibration and 10.3 ± 8.3 s for the second period. There was no difference in latency between sides of vibration (*F*(1, 16) = 0.61, *p* = 0.45) or conditions (control tasks and PKAR periods) (*F*(2, 32) = 2.4, *p* = 0.11) and no interaction between sides of vibration and conditions (*F*(2, 32) = 1.66, *p* = 0.2).

Figure 3(b) shows the result of the analysis made to explore whether vibration significantly modulated the PKAR features. We averaged the angular velocities in selected time intervals. These intervals ranged from 150 s to 180 s (PKAR, no vibration), from 220 s to 280 s (1st period of vibration), from 300 s to 360 s (no vibration), from 440 s to 500 s (2nd period of vibration), and from 540 s to 600 s (no vibration). The values of the individual subjects that entered the averaging procedure were the mean values of the angular velocities calculated within a 1 min period centered on the selected time intervals during the reference PKAR and the two PKAR+vibration sessions. The shorter duration of the analyzed periods with respect to the 2 min duration of the vibration intervals allowed for the changes in angular velocity to reach the plateau.

Vibration almost blocked the PKAR when it was administered on the right side, i.e., when the vibration alone would cause a CCW rotation, opposite to the PKAR CW rotation (some subjects even reversed the PKAR rotation sense). The fall in angular velocity was of 11.5°/s and 8.5°/s in the first and second intervals of vibration, respectively. The amplitude of the effect was less striking when the vibration was administered on the left side. In this case, the CW PKAR rotation speed increased (5°/s and 5.7°/s in the two intervals), but the increments were smaller in absolute value than in the case of the contralateral vibration. This difference was however not significant, for either the first or for the second vibration period. Repeated measures ANOVA (conditions, intervals) showed that angular velocities diminished as a function of time elapsed from the peak of the PKAR. All conditions collapsed, and there was a significant difference between the five intervals (*F*(4, 64) = 18.56, *p* < 0.001). The three conditions were different as well from each other (*F*(2, 32) = 7.09, *p* < 0.005). There was a significant interaction between conditions and intervals (*F*(8,128) = 7.44, *p* < 0.001). Post hoc test indicated there were no differences between the three conditions (reference PKAR, PKAR+right vibration, and PKAR+left vibration) in the two intervals free from vibration (*p* > 0.2, for all comparisons). During the intervals with vibration (right or left), the mean values of the angular velocities were different with respect to the corresponding reference PKAR intervals (*p* < 0.05, for all comparisons), being either lower during right vibration or higher during left vibration. This occurred in spite of the steady decrease in PKAR angular velocity from the first to the fifth interval.

### 3.3. Cadence and Foot Angle

**Figure 4:**
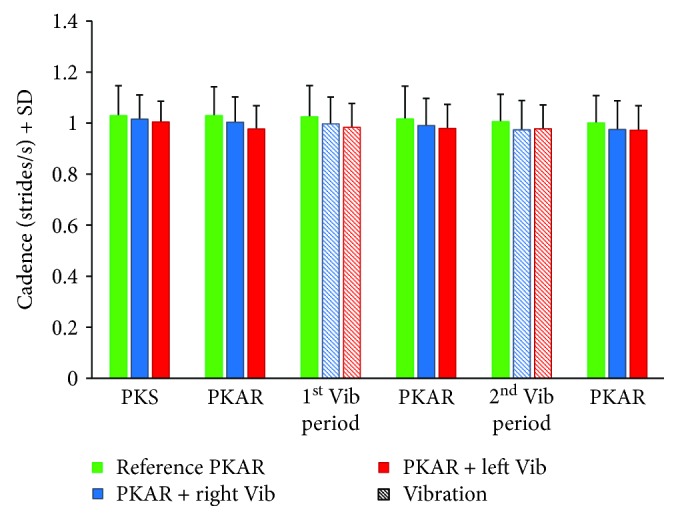
Mean cadence is reported for the last minute of the PKS period and for the different PKAR periods that had been considered for the rotation velocity, without and with vibration. Green bars refer to reference PKAR, blue bars refer to PKAR with vibration applied to lumbar muscle of the right side, and red bars refer to PKAR with left-side lumbar muscle vibration. Striped bars refer to the periods during which vibration was present. There is no difference in cadence between the three conditions.

Figure 4 shows the mean cadence calculated during the PKS and PKAR periods in the same time intervals considered for the previous analysis. Repeated measures ANOVA (conditions, intervals) showed that cadence did not differ between the three conditions (reference PKAR, PKAR+right vibration, and PKAR+left vibration) (*F*(2, 32) = 1.86, *p* = 0.17). There was a significant difference between intervals (*F*(5, 80) = 4.65, *p* < 0.001) but no significant interaction between conditions and intervals (*F*(10,160) = 1.24, *p* = 0.27).

**Figure 5:**
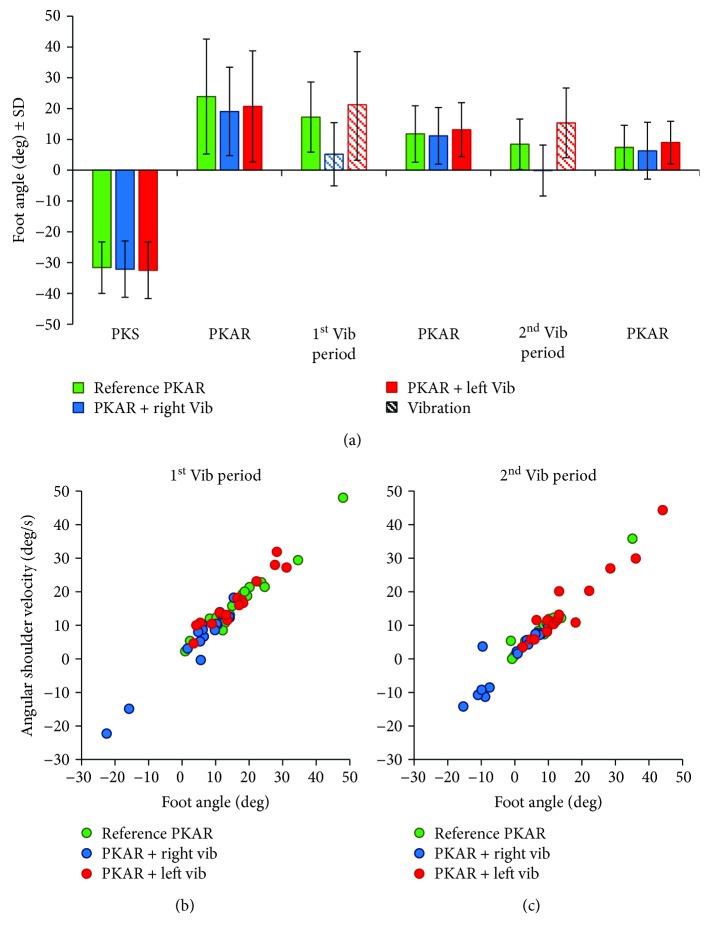
Step yaw angle. (a) Mean step yaw angle calculated for the right foot during the last minute of the PKS and during the PKAR (without and with vibration at different time intervals—the same intervals considered for the calculation of the angular velocity). Green bars refer to reference PKAR, blue bars refer to PKAR with right-side vibration, and red bars refer to PKAR with left-side vibration. Striped bars refer to the vibration periods. There is a significant difference in the foot yaw angle between reference PKAR and PKAR with vibration in both periods in which vibration was present. (b, c) Relationships between mean foot angle and body rotation velocity during the first (b) and second (c) periods of vibration. Each dot corresponds to a subject. Green dots refer to PKAR without vibration, blue dots refer to PKAR with right-side vibration, and red dots refer to PKAR with left-side vibration. There is a good relationship between yaw foot angle and body rotation velocity (*p* < 0.001 for all the regression lines across conditions and vibration periods).

Figure 5(a) shows the mean step yaw angles of the right foot (again calculated for the same intervals of the previous analysis). There was a significant difference between conditions (*F*(2, 32) = 8.31, *p* < 0.05) and intervals (*F*(5, 80) = 116.1, *p* < 0.001) and a significant interaction between condition and intervals (*F*(10,160) = 6.24, *p* < 0.001). The foot yaw angle diminished as a function of time, much as occurred with the shoulder angular velocity. The post hoc analysis showed that there were no differences in foot yaw angle between the three conditions during the periods without vibration (*p* > 0.1 for all comparisons), but there were significant differences in foot yaw angle between PKAR and PKAR with vibration (right or left) in the periods in which the vibration was present (*p* < 0.05 for all comparisons). When vibration was administered to either side of the trunk, the yaw foot angle diminished (or increased) with respect to the angle calculated on the same time period of the PKAR without vibration, in keeping with the decrease (or increase) of the velocity of body angular rotation. Figures 5(b) and 5(c) show the good relationship between yaw foot angle and the shoulder rotation velocity (*p* < 0.001 for all the regression lines) across the three conditions, in both the first and second vibration periods. Overall, foot yaw angle was consistent with the corresponding whole-body rotation velocity, in spite of large interindividual differences in angular velocity.

## 4. Discussion

Stepping in place on a rotating treadmill for an extended period of time produces a clear-cut after-effect. Whole-body rotation continues when subjects keep on stepping eyes closed after treadmill stop. This podokinetic after-rotation (PKAR) ensues almost immediately, reaches a peak of angular velocity very soon, within few seconds or steps, and decays exponentially over time [18]. It is as if the neural centers producing the counter-rotation effort while stepping on the rotatory platform had been coiled up like a spring, which then unwinds progressively and decrementally, so that in some minutes, the spring becomes completely slack.

Body rotation while stepping can be obviously voluntarily produced, but it can be elicited by a proprioceptive stimulation of axial muscles as well (consisting in a vibratory stimulus at a frequency known to activate the muscle spindles, see, e.g., [29–31]). This procedure has a somewhat less predictable outcome than the podokinetic stimulation. With vibration, rotation may not ensue immediately in all cases when subjects step in place [1]. In the present study, across subjects, the effects began in a time interval ranging from a few seconds to less than half a minute. Similar latencies were observed when vibration was administered during PKAR. Further, vibration had no after-effects, so that on switching the vibrator off, the rotation soon disappeared. After-effects have been shown for limb muscle vibration [32], but not for axial muscle vibration. Importantly, during stepping in place or walking, body rotation is not attributable to adaptation to a postural state disturbed by vibration. This has been convincingly shown by Bove et al. [1] and Courtine et al. [16]. Osler and Reynolds [23] showed that this is true also for the body rotation while stepping in place associated with the PKAR, since no relationship was found between rotation velocity and trunk reorientation.

### 4.1. Algebraical Summation of Podokinetic After-Rotation and Rotation Induced by Asymmetric Proprioceptive Stimulation

Here, we have designed a simple protocol to check the hypothesis that a single process is shared by both podokinetic stimulation after-effect (the PKAR) and vibration. Since both responses have a clear-cut directional effect, applying vibration during PKAR would enhance or reduce the body rotation velocity depending on the selected side of stimulation. As a matter of fact, we have seen a facilitation of the ongoing PKAR when the vibration was applied on the side opposite to that toward which the body rotated while stepping during the podokinetic after-effect (left side in our case). Conversely, when the vibration was administered to the side toward which the body rotated (right side), the rotation diminished or even reversed. Hence, the “rotatory” effect of the vibration algebraically added to the PKAR rotation.

When the vibration was delivered at a time at which the podokinetic after-rotation was intense, the vibration-induced increment of the podokinetic after-rotation appeared to be smaller than the vibration-induced decrement observed with the administration of contralateral vibration. The differences were not significant, due to the large intersubject variability in both PKAR alone and in the vibration effects, possibly depending on the effectiveness of the actual mechanical action of the vibrators on the muscle bellies. Definitely, though, when the vibration effect was adding to the PKAR, the angular velocity hardly bypassed the maximum peak velocity of the PKAR itself, as if a sort of occlusion occurred. The facilitation became relatively larger as time progressed and podokinetic after-rotation became weaker.

Remarkably, at the onset of vibrations during the PKAR period, regardless of the absolute entity of the effect, the emergence of the effect was rapid (less than 10 s), not different from what occurred under the “control” stepping in place condition, and similar to what occurs during walking [15, 16]. The vibration effect appeared to be simply additive (in other words, the increment or decrement of the angular velocity during vibration accompanied the decrease in PKAR). Then, when the vibration stopped, the podokinetic after-effect fully resumed, and body rotation returned to the value expected for that particular time period. Since the PKAR exponentially decreased over time, the rotation velocity at the end of the vibration periods was the same it would have been if the vibration had not been administered.

### 4.2. Resilience of the PKAR

Vibration did not persistently disrupt the slow unwinding of the coiled spring. This finding is parallel to that described by Falvo et al. [26], who showed interaction with the PKAR by vision and touch. When vision was allowed during the podokinetic after-effect, the rotation immediately ceased, to reinstate immediately after closing the eyes [26, 33]. The podokinetic after-rotation is therefore robust to the vibratory perturbation in spite of its relatively long duration and progressive weakening. Repetition of vibration bouts is also ineffective in modifying the ongoing podokinetic after-rotation. Difference with Falvo et al. [26] is that our proprioceptive stimulation not only stopped the ongoing PKAR like visual or haptic inputs but also consistently increased (or decreased) the extent of the PKAR rotation. Since vibration effect was a quasi-algebraical sum of the PKAR and the vibration-induced rotation, a simplistic interpretation would be that a neural center is producing both rotations, and its final effect depends on the interaction between the stored activity and the perturbing input from the vibratory stimulation.

It seems that a single process is put into action by both podokinetic stimulation and vibration. The algebraical summation would speak for only one “rotation center,” accessed by both the PKS and the vibration inputs. However, the slow process responsible for building up and storing the PKS-induced tendency to rotate (i.e., the PKAR) would not be occurring within the same “rotation center” promptly activated by the vibration, but would occur elsewhere in the central nervous system and would send its influence (slowly decaying over time) to this “center.” Conversely, the proprioceptive volleys would directly affect this same center, favoring its functioning CW or CCW depending on the vibrated side. We do not believe that the vibration-triggered volley collides with the very same neural circuits that store the PKS effect and produce the PKAR. The vibration did not disrupt PKAR. Off vibration, PKAR resumed its original course, so that it was perfectly superimposed to the profile of the PKAR recorded under the no-vibration condition. It is as if vibration exerted no effect on the very process of slowly unwinding the spring, previously coiled up during the PKS, at the same time that it had a striking effect on the expression of the podokinetic motor after-effect.

### 4.3. Would Modulation of the Straight Ahead Explain the Interaction of PKAR and Vibration?

All subjects were questioned about their feelings after the experiment. Interestingly, none of them had noticed the rotatory body motion while stepping, be it either for the PKAR or for the vibration-induced rotations (or even for the changes in rotation sense), in spite of the vibration itself being clearly perceived. No sensation of movement was elicited [16, 34, 35]. Since the labyrinth elicits clear-cut self-motion perception [36–38], the interpretation of our findings would be in keeping with the conclusions by Earhart et al. [28] and Sozzi and Schieppati [20] that PKAR is mediated primarily by somatosensory information, while vestibular inputs may not be needed for its expression. This seems to be true even when vibration encroaches onto the PKAR and dramatically changes its amplitude or rotation sense. Likewise, the effect of vibration applied to the lateral neck muscle [16, 39] is not mediated by vestibular activation [40]. This would be all the more true for vibration of the trunk muscles at lumbar level, as in the present study, where distance between vibrator and mastoid bone does not leave space to doubt.

Scott et al. [41] showed that PKS produced a shift in the subjective straight ahead, and that the effect was direction-specific, i.e., dependent on the platform rotation sense during PKS. Subjects who stepped in place on the platform rotating clockwise (and therefore exerted a counterclockwise—leftward—effort in order to keep constant their orientation in space) pointed to the left of straight ahead and vice versa. As to vibration is concerned, many past and recent investigations report clear-cut effects of axial muscle vibration on the perception of the vestibular-evoked self-motion [42] and straight ahead [43, 44] (see for a review Pettorossi and Schieppati [45]). In spite of uncertainties likely connected with the high variability in the ability to perceive and report motion perception across subjects or to differences in the location of vibration spots, or both, it appears to be established that axial (neck) muscle vibration produced a horizontal deviation of the perceived straight-ahead perception toward the side of stimulation [46, 47].

The algebraical summation of PKAR and vibration effects is not peculiar or odd. Under definitely different ecological conditions but with a research question bordering our own, Fitzpatrick et al. [48] reported that galvanic vestibular stimulation (GVS) cancelled the perception of rotation reported by supine subjects rotating in yaw around their labyrinth when the GVS-induced motion perception was incongruent with the rotation. When the vestibular signal of rotation and the actual body yaw rotation were congruent, subjects reported higher body yaw rotation velocity. Compatible findings were reported by Deshpande and Patla [49].

Perhaps, the PKS gradually modifies the straight ahead through the rhythmic forced incitement of pelvis-on-leg voluntary rotation during stepping in place, implying continuous asymmetric volleys from the spindles of the acting muscles. Then, when the PKS effect slowly vanishes during the PKAR period, the straight ahead gradually returns to the default position. It is during this period that asymmetric vibration intrudes into the circuits responsible for building the straight ahead and modifies it (shifting it either side). This supposition would be supported by the findings of Duclos et al. [50], who showed very similar brain activation patterns in supplementary motor area and cerebellum under both postvibration and postcontraction periods (following voluntary tonic contraction, see Section 4.4).

### 4.4. Analogies between PKAR and the Kohnstamm Phenomenon

The findings mentioned above, and the changes in angular velocity (either way), would suggest a combined action of the PKAR command and of the vibration at the level of some executive circuit. It would be tempting to identify this center with that responsible for the Kohnstamm phenomenon (KP) [32, 51, 52], even if, admittedly, we do not have enough data for a complete comparison. This is a slowly mounting involuntary contraction of one or more muscles after a preceding protracted period of voluntary contraction of the same muscles. In the context of the present study, we would note that Ivanenko et al. [53] asked standing subjects to oppose a sustained rotational torque applied to the pelvis either in the clockwise or in the counterclockwise direction for half a minute. Then, subjects asked to walk eyes closed travelled a curved trajectory in the direction of the preceding torsion, without being aware of the effect. In our case, the rotator muscles of the legs, alternately active for minutes during the PKS, would be the source of the information ultimately producing sort of KP at pelvis level. Interestingly, when Ivanenko et al. [53] asked the subjects to step in place, no rotational component was observed. However, this was likely because the conditioning period (30 s) was too short-lasting compared with the 10 min or more necessary for the PKS to produce a full-blown PKAR. Further, the lower-limb rotator muscles, certainly active during both the PKS and the PKAR, were likely not called into action to any large extent by their maneuver.

In the present investigation, the PKAR was easily cancelled (or enhanced) by unilateral vibration of trunk muscles. Apparently, the KP is very sensitive to any movement-related information. Ghosh et al. [54] asked subjects to bring the arm down during KP voluntarily, and the involuntary arm abduction ceased. When this voluntary effort was withdrawn, the involuntary arm lift resumed, much as it occurred in our hands in the PKAR plus vibration conditions. Past research suggested already that the egocentric, body-centered coordinate system that determines our body position with respect to the environment is highly sensitive to voluntary movement and proprioception [46]. In this connection, it seems not inappropriate to mention that both straight walking and curved walking depend on a robust plant [55, 56], and that minor modulations would be more than enough for changing one into the other behavior.

## 5. Conclusion

The present findings are in line with several papers in the literature that have investigated our orientation in space, under both normal and unhealthy states (see, e.g., [57–59]). They add new information about the strong interaction of asymmetric proprioceptive input from the body axis with the podokinetic after-rotation. However, it is still problematic to define with confidence the brain region(s) responsible for this all-important function. One open question is whether the algebraical summation of the information producing the PKAR and that from the vibration-induced input exclusively occurs at supraspinal, possibly cortical level [60, 61] or brainstem and cerebellar level [24, 62, 63]. In the former case, our stepping body would follow a continuously mutable sensed heading direction, and in the latter, the asymmetries of PKS and vibration would exert their effects, or part of this, on the brainstem centers [64] able to store locomotor adaptations and ultimately affecting the spinal centers mediating the adapted locomotion. Certainly, either or both interventions investigated here (PKS and vibration) might be considered when having in mind to design a training protocol aimed at rehabilitating gait, with emphasis on curved walking in either or both hemiplegic and Parkinsonian patients [25, 61, 65, 66].

## Data Availability

The data used to support the findings of this study are included within the article.
